# On the Performance of Efficient Channel Estimation Strategies for Hybrid Millimeter Wave MIMO System

**DOI:** 10.3390/e22101121

**Published:** 2020-10-03

**Authors:** Prateek Saurabh Srivastav, Lan Chen, Arfan Haider Wahla

**Affiliations:** 1 Institute of Microelectronics of Chinese Academy of Sciences, Beijing 100029, China; irfan.hayd@gmail.com; 2 School of Electronics, Electrical and Communication, University of Chinese Academy of Sciences, Beijing 100049, China

**Keywords:** millimeter wave, MIMO, beamforming, ADMM, convex optimization, channel estimation, ADMM

## Abstract

Millimeter wave (mmWave) relying upon the multiple output multiple input (MIMO) is a new potential candidate for fulfilling the huge emerging bandwidth requirements. Due to the short wavelength and the complicated hardware architecture of mmWave MIMO systems, the conventional estimation strategies based on the individual exploitation of sparsity or low rank properties are no longer efficient and hence more modern and advance estimation strategies are required to recapture the targeted channel matrix. Therefore, in this paper, we proposed a novel channel estimation strategy based on the symmetrical version of alternating direction methods of multipliers (S-ADMM), which exploits the sparsity and low rank property of channel altogether in a symmetrical manner. In S-ADMM, at each iteration, the Lagrange multipliers are updated twice which results symmetrical handling of all of the available variables in optimization problem. To validate the proposed algorithm, numerous computer simulations have been carried out which straightforwardly depicts that the S-ADMM performed well in terms of convergence as compared to other benchmark algorithms and also able to provide global optimal solutions for the strictly convex mmWave joint channel estimation optimization problem.

## 1. Introduction

The standard wireless communication system is exhausted due to the large number of users as well as by the high data speed demands [1]. Millimeter waves (mmWaves) represent a promising candidate with a large amount of unused bandwidth and the ability to support millions of devices at once [2]. mmWaves have very short wavelengths therefore the hardware structure of mmWave relying upon a multiple input multiple output (MIMO) system is unlike the conventional sub 6 Ghz wireless communication system [3]. The smaller wavelength of mmWaves make them perfectly compatible for multi-user MIMO systems accompanied by large antenna arrays. Since the mmWave frequencies are highly directional as compared to lower frequencies therefore they can precisely handle large antenna arrays during the transmission and reception process and a beamforming strategy is required for mmWave MIMO systems. Here the term “beamforming” conventionally refers to the set of smart antenna arrays. Beamforming restricts transmitted signals to a particular desired receiving antenna element available in an antenna array at the receiver’s end. Consequently, for achieving the high array, diversity and multiplexing gain, beamforming plays an important role in mmWave MIMO systems [4]. Generally, three kind of beamforming techniques—analog beamforming (ABF), digital beamforming (DBF) and hybrid beamforming (HBF)—are used. ABF steers the ultra linear array (ULA) output using a single RF chain and phase shifters [5]. However, the analog structure cannot be equipped with multiplexing advantages. On the contrary, DBF offers the flexibility needed to support multi-stream data transmission, but the hardware is expensive and power consuming as it consists of separate RF chains (with ADC/DAC) for every antenna element in the uniform linear array (ULA) [5]. Therefore, due to the aforementioned limitations, ABF and DBF are not considered suitable for mmWave MIMO systems and hence the HBF technique which combines both analog and digital beamforming architectures and provides a better trade-off between cost/complexity and spectral efficiency is used for enabling the communication in mmWave MIMO systems [6,7].

In any wireless communication system, efficient estimation of the wireless channels at the receiver’s end is the only way to ensure the quality of transmitted symbols. Apparently, channel estimation is a vulnerable task for any type of wireless communication system. For achieving the potential advantages of mmWave MIMO system, obtaining the accurate knowledge of channel state information (CSI) is critically important. As the mmWave MIMO system’s operating regime and hardware constraints are different than those of conventional wireless communication systems therefore new channel estimation strategies are needed. There are several popular channel estimation techniques for mmWave MIMO systems are already available in the research domain. These strategies are based on compressive sensing (CS) where the sparsity of the channel is exploited in the angular or beamspace domain [8,9,10,11,12,13,14] or due to the narrow angle spread of individual clusters, the low rank properties of channel covariance matrices (CCMs) are being investigated [15]. The most common approach for the estimation of CSI is to consider it as a CS problem [16]. In [17,18] the estimation techniques require receiver feedback which can further increase the pilot demands and reduce the spectral efficiency of the system. Statics dictionaries and beam training methods are also discussed in [13,19,20,21,22]. These methods do not require receiver feedback and they exploit the static dictionaries of the channel matrix which usually have the information of angle of arrival (AoA) and angle of departure (AoD), but for a larger number of training overheads, the static dictionaries generate errors related to discretization and power leakage. One of the most popular CS approaches is orthogonal matching pursuit (OMP), described in [23]. Exploitation of angle information for sparse channel estimation is described in [18] in which a fast discrete Fourier transform (DFT)-based spatial rotation algorithm is designed to contemplate most of the channel power on limited DFT grids and efficiently obtain the angle information for both frequency division duplex (FDD) as well as time division duplex (TDD) systems [24]. Specifically, the array signal processing-based channel estimation scheme, where the angle information of the user is exploited to simplify channel estimation is illustrated in [24]. A CCM-based approach are described in [25]. In any typical scattering atmosphere, [25] demonstrates the low-rank feature of the CCM’s in mmWave communications and to curtail the effective dimensions of the channel, it elaborates the collective spatial division multiplexing algorithm. The channel estimation problem is assigned as a quadratic semidefinite programming (SDP) problem where the low-rank structure of the CCMs are used and solved by using a polynomial SDP method is interpreted in [26]. In [27], a virtual channel with common sparsity because users are sharing the same local scatters, is explained in which the information of unitary dictionary matrix is available at a base station (BS). A comprehensive study on signal processing techniques used for mmWave MIMO communications is briefly explained in [28].

Alternating direction method of multipliers (ADMM) was recently proposed in [29] and it has attracted extensive attention due to its simple implementation. It is widely used in distributed machine learning [30], image processing [31], statistical signal processing [32] and many more fields. ADMM breaks any complicated optimization problem into several small subproblems therefore one can derive the optimal solutions very easily [29]. ADMM is used for the narrowband and wideband channel estimation of mmWave MIMO system by exploiting the sparsity and low rank properties of channel were jointly exploited for efficient CSI estimation in [16,33]. An extended version of ADMM (Ex-ADMM) with a Fortin and Glowinski’s constant (i.e., the relaxation parameter) is also used for the narrowband channel estimation of mmWave MIMO systems in [34]. A symmetric version of ADMM (S-ADMM) came into the research domain very recently [35,36]. Within this scheme, the Lagrange multipliers are updated twice in a symmetrical manner [35]. The studies indicates that the convergence of S-ADMM with larger step sizes, can be enlarged with the help of Fortin and Glowinski’s constant [35]. The symmetrical formation of ADMM also results an enhancement in the overall performance of the system.

The contributions of this paper can be summarized as follows:

(1) A novel S-ADMM based channel estimation scheme for the estimation of mmWave channels relying on a MIMO system is proposed. After updating the Lagrangian multipliers twice, a symmetrical version of ADMM can optimized the intermediate and essential variables in a symmetrical order. In addition with the Fortin and Glowinski’s constant which is generally known as a relaxation parameter, the convergence of the algorithm can be enhanced. In this paper, the overrelaxed version of relaxation parameter have been considered for simulation and experiments.
(2) To explain the superiority of the proposed scheme, various different popular start-of-art schemes namely, OMP [13] Vector Message Approximation Passing (VAMP) [37], Ex-ADMM [34], ADMM [33], Block Orthogonal Matching Pursuit (BOMP) [38], Generalized Approximate Message Passing with Gaussian Mixture (GAMP-GM) [39,40] and Singular Value Thresholding (SVT) [41] have been considered for the comparison. Furthermore, the eminence of the proposed scheme is explained in terms of normalized mean squared error (NMSE), achievable spectral efficiency (ASE), convergence, effect on the number of scatterers and the number of possible paths.

The rest of the paper is assembled as follows: Section 2 described the System Model used for various studies within this paper. The problem formulation for the channel estimation of mmWave MIMO system along with a detailed description of proposed scheme followed by the algorithm terminologies and complexity analysis is depicted in Section 3. Simulation and results are explained in Section 4 and finally, last but not least, conclusion remarks are elucidated in Section 5.

**Notation:** The notation used within this paper is described in Table 1.

## 2. System Model

**Figure 1:**
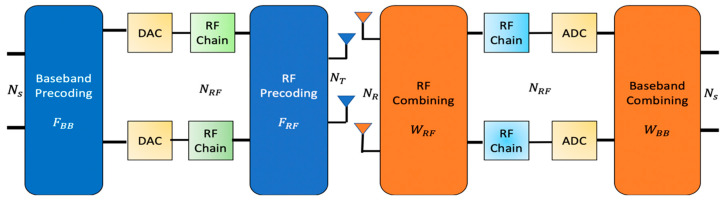
Hybrid mmWave MIMO architecture.

A hybrid mmWave MIMO system is a constellation of two continuous segments namely, a digital MIMO baseband $F_{\mathrm{BB}\;}\in \;{\mathbb{C}}^{N_{\mathrm{RF}}\times N_{S}}$ and an analog RF precoder $F_{\mathrm{RF}\;}\in \;{\mathbb{C}}^{N_{T}\times N_{\mathrm{RF}\;}}$ at the transmitter section while at the receiver section it has two successive joint segments of a RF combiner $W_{\mathrm{RF}\;}\in \;{\mathbb{C}}^{N_{R}\times N_{\mathrm{RF}\;}}$ and a baseband combiner section $W_{\mathrm{BB}\;}\in \;{\mathbb{C}}^{N_{\mathrm{RF}}\times N_{S\;}}.$ For our studies, the HBF structure explained in [19] is adopted. Wherein, a point to point $N_{R}\times N_{T}$ mmWave MIMO system, equipped with $N_{T}$ transmit and $N_{R}$ receive antennas at base station (BS) and mobile station (MS), respectively, is considered [42] as depicted in Figure 1.

At the transmitter and receiver section, this system is provided with $N_{S}$ parallel data streams and radio frequency (RF) chains, such that $N_{\mathrm{RF}}\leq \min\left(N_{T},N_{R}\right)$ [43,44]. The transmitter section is furnished with $N_{\mathrm{RF}\;}$ RF chains in such a way that $N_{S}<N_{\mathrm{RF}\;}<N_{T}$. For initiating the communication, the transmitter employed $N_{T}^{\mathrm{Beam}}\leq N_{T}$ pilot beam patterns, denoted as $\{f_{a}\in$ ${\mathbb{C}}^{N_{T}\times 1}:||f_{a}||{}_{2}^{2}\;=\;1\}$ whereas, at the receiver end, the receiver employed $N_{R}^{\mathrm{Beam}}\leq N_{R}$ pilot beam patterns, denoted as $\{w_{b}\in$ ${\mathbb{C}}^{N_{R}\times 1}:||w_{b}||{}_{2}^{2}=1$ [13], where a and b are the transmitter’s training precoding vector and receiver’s training combining vector, respectively [13].

After the initial transmission, the received signal matrix Y at the receiver’s end can be determined as:

(1) $$Y\;={\;W}^{H}\mathrm{AFX}+Q$$

where, the received signal matrix is the combination of different received vectors, i.e., $Y≜\left[y_{1},\ldots .,{\;y}_{N_{T}^{\mathrm{Beam}}}\right]\in {\mathbb{C}}^{N_{R}^{\mathrm{Beam}}\times N_{T}^{\mathrm{Beam}}}$, alike Y the combining matrix W and precoding matrix F is also representing by the set of different combining and precoding vectors i.e., $W≜\left[w_{1},\ldots .,{\;w}_{N_{R}^{\mathrm{Beam}}}\right]\in {\mathbb{C}}^{N_{R}\times N_{R}^{\mathrm{Beam}}}$ and $F≜\left[f_{1},\ldots .,{\;f}_{N_{T}^{\mathrm{Beam}}}\right]\in {\mathbb{C}}^{N_{T}\times N_{T}^{\mathrm{Beam}}}$, respectively. Here, $X\in {\mathbb{C}}^{N_{T}^{\mathrm{Beam}}\times N_{T}^{\mathrm{Beam}}}$ is the set of transmitted vectors, A is the channel matrix and $Q\in {\mathbb{C}}^{N_{R}^{\mathrm{Beam}}\times N_{T}^{\mathrm{Beam}}}$ are independent and identically distributed (I.I.D) complex additive white gaussian noise (AWGN), with zero mean and $\sigma_{q}^{2}\;\mathrm{variance}\;CN\left(0,\sigma_{q}^{2}\right)$ [34]. For the simplicity of the system, let’s consider that the all pilot symbols are identically similar, therefore, one can assume that $X\;=\;\sqrt{P_{t}}I_{N_{T}^{\mathrm{Beam}}}$. Here $P_{t}$ expressed the average transmitted pilot power [13,18].

As it is clear from the HBF architecture described in [19], Equation (1) can be re-written on the basis of decomposition of W and F, i.e., $F\;={\;F}_{\mathrm{BB}}F_{\mathrm{RF}},$ and $W\;={\;W}_{\mathrm{RF}}W_{\mathrm{BB}}$. Therefore:

(2) $$Y≜\;\sqrt{P_{t}}{\;W}_{\mathrm{BB}\;}{}^{H}W_{\mathrm{RF}\;}{}^{H}{\mathrm{AF}}_{\mathrm{BB}}F_{\mathrm{RF}}+Q\;Y≜\;\sqrt{P_{t}}{\;W}^{H}\mathrm{AF}+Q$$

where, $F_{\mathrm{RF}\;}\in \;{\mathbb{C}}^{N_{T}\times N_{T}}$ and $W_{\mathrm{RF}\;}\in \;{\mathbb{C}}^{N_{R}\times N_{R\;}}$ are the transmitted and received beamforming matrices, respectively. $F_{\mathrm{BB}\;}\in \;{\mathbb{C}}^{N_{T}\times N_{T}^{\mathrm{Beam}}}$ and $W_{\mathrm{BB}\;}\in \;{\mathbb{C}}^{N_{R}\times N_{R}^{\mathrm{Beam}}}$ are the transmitted and received baseband processing matrices, respectively [19]. W is the combiner, such that $\;W\in {\left\{0,1\right\}}^{N_{R}}$, and F is the precoder, such that $\;F\in {\left\{0,1\right\}}^{N_{T}}$ [19].

According to the geometric virtual (GV) model of mmWave MIMO system explained in [12,18], Equation (2) can be further elaborated as:

(3) $$A≜\overset{L_{p}}{\underset{l=1}{\sum }}\alpha_{l}\;d_{R}(\Phi_{R}^{\left(l\right)},\theta_{R}^{\left(l\right)})d_{T}^{H}\left(\Phi_{T}^{\left(l\right)},\theta_{T}^{\left(l\right)}\right)$$

where, $L_{p}$ denotes the total number of propagation paths, $\alpha_{l}$ expressing the complex channel gain of the *l-th* path, and it can be obtained from the random complex Gaussian distributions, and $CN\left(\frac{0,1}{2}\right)$. $d_{T}^{H}\left(\Phi_{T}^{\left(l\right)},\theta_{T}^{\left(l\right)}\right)\in {\mathbb{C}}^{N_{T}}$ and $d_{R}\left(\Phi_{R}^{\left(l\right)},\theta_{R}^{\left(l\right)}\right)\in {\mathbb{C}}^{N_{R}}$ are the array response vectors (ARV) at the transmitters and receivers, respectively [34] (see the references therein). $\Phi_{T}^{\left(l\right)},\theta_{T}^{\left(l\right)}$ and $\Phi_{R}^{\left(l\right)},\theta_{R}^{\left(l\right)}$ are the elevation and azimuth AoA and AoD angles at the transmitters and receivers, respectively [34] (see the references therein). The elevation and azimuth AoA and AoD angles can be produced by uniform Laplacian distributions, allocated within the range of 0 and 2π.

According to [6,45], ARV of a ULA can be expressed as:

(4) $$d\left(\theta \right)\;=\;\frac{1}{\sqrt{N}}{\left[1,e^{-j\frac{2\pi }{\lambda }\mathrm{kcos}\left(\theta \right)},\ldots .{\;e}^{-j\frac{2\pi }{\lambda }\left(N-1\right)\mathrm{kcos}\left(\theta \right)}\right]}^{T}$$

where, the wavelength is denoted by λ, k is the spacing between the antennas and the ARV’s even function is θ.

Based on the virtual beamspace representation model, Equation (4) can be rewritten in matrix decomposition form [46,47]. Therefore, the channel matrix A can be expressed as:

(5) $$A\;={\;D}_{R}{\mathrm{ZD}}_{T}^{H}$$

where the receiver’s and transmitter’s ARV’s in terms of unitary matrices are $D_{R}\;\in$ ${\mathbb{C}}^{N_{R}\times N_{R}}$ and $D_{T}\;\in {\mathbb{C}}^{N_{T}\times N_{T}}$ [46], respectively and these are expressed as $D_{R}≜\left[d_{R}\;\left(\Phi_{1},\theta_{1}\right),d_{R}\left(\Phi_{2},\theta_{2}\right)\ldots \ldots .d_{R}\left(\Phi_{L_{p}},\theta_{L_{p}}\right)\right]{\;\mathrm{and}\;D}_{T}≜\left[d_{T}\left(\Phi_{1},\theta_{1}\right),d_{T}\left(\Phi_{2},\theta_{2}\right)\ldots \ldots .d_{T}\left(\Phi_{L_{p}},\theta_{L_{p}}\right)\right].$

From the matrix property, $D_{R}^{H}D_{R}\;={\;I}_{N_{R}}$ and $D_{T}^{H}D_{T}\;={\;I}_{N_{T}}$ are N × N identity matrix $I_{N}$. In Equation (5), Z has the several virtual channel gains of higher amplitude, therefore it is known as sparse matrix and Z ∈ ${\mathbb{C}}^{N_{R}\times N_{T}}$.

## 3. Proposed Channel Estimation Scheme for mmWave MIMO System

In this section, the optimization problem followed by the solution obtained through proposed S-ADMM based scheme is described in detail. Additionally, the computational complexity as well as the algorithm terminology is also discussed briefly.

### 3.1. Problem Formulation for mmWave MIMO System

Partially observed data are very helpful for completing the missing entries of a low rank matrix [48,49] therefore, to formulate the optimization problem for the channel estimation of mmWave MIMO system, Equation (5) is split into a decomposed version such as $A\;={\;D}_{R}{\mathrm{CD}}_{T}^{H}$, where C defines the submatrix of Z and it has the information of subsampled values of Z.

Thus, to recover the CSI matrix A, the joint optimization problem can be therefore illustrated as:

(6) $$\underset{A,C}{\mathrm{minmize}}\;\tau_{A}{\left||A|\right|}_{*}+\tau_{C}{\left||C|\right|}_{1}\;\mathrm{Subject}\;\mathrm{to}\;\Psi \circ A\;={\;A}_{\Psi }\;\;\mathrm{and}\;A\;=\;D_{R}{\mathrm{CD}}_{T}^{H}$$

In the cost function described in Equation (6), $D_{R}{\;\mathrm{and}\;D}_{T}$ are treated as the side information of matrix C. These informations are able complete the missing entries of low rank matrix A. The nuclear norm on matrix A ensured its low rankness and the $l_{1}-\mathrm{norm}$ on C ensured the sparsity on C. $\tau_{A}$ and $\tau_{C}$ are known as the weighting factors and it generally rely upon the number of propagation path. These weighting factors are always assumed to be a positive number i.e., $\tau_{A}$, $\tau_{C}>0$ [48].

### 3.2. Proposed S-ADMM Scheme for mmWave MIMO System

The optimization problem described in Equation (6) is clearly a two objective strict convex function. Thus, solution of Equation (6) can be obtained by numerous methods. Generally, alternating optimization techniques (AOTs) are the best selection for solving Equation (6). ADMM [29] is the one of the best known AOT’s for solving the strict convex problems. Therefore, to get the optimal solutions of Equation (6), reformulate it and introduced two auxiliary matrices, $B\in \;{\mathbb{C}}^{N_{R}\times N_{T}}$ and $D≜B-D_{R}{\mathrm{CD}}_{T}^{H}$. Hence, the new targeted optimization problem can be expressed as:

(7) $$\underset{A,B,C,D}{\mathrm{minmize}}\;\tau_{A}{\left||A|\right|}_{*}+\tau_{C}{\left||C|\right|}_{1}+\frac{1}{2}{\left||D|\right|}_{F}^{2}+\frac{1}{2}||\Psi \circ B-A_{\Psi }||{}_{F}^{2}\;\mathrm{Subject}\;\mathrm{to}\;A\;=\;B\;\mathrm{and}\;D\;=\;B-D_{R}{\mathrm{CD}}_{T}^{H}$$

The new cost function defined in Equation (7) contains different information related to different parameters. The first term holds the side information of low rank matrix A. The second term contains the information of subsampled virtual channel gain. Third and fourth term have the statistics of discretization errors and AWGN noise, respectively. Subsequently, Equation (7) can be written under the augmented Lagrangian function (ALF) as follows:

(8) $$\begin{array}{cc} ℒ\left(A,B,C,D,Z_{1},Z_{2}\right)\\ & ≜\tau_{A}{\left||A|\right|}_{*}+\tau_{C}{\left||C|\right|}_{1}+\frac{1}{2}{\left||D|\right|}_{F}^{2}+\frac{1}{2}||\Psi \circ B-A_{\Psi }||{}_{F}^{2}+\mathrm{tr}\left(Z_{1}^{H}\left(A-B\right)\right)\\ & +\frac{\rho }{2}||A-B||{}_{F}^{2}+\mathrm{tr}\left(Z_{2}^{H}\left(B-D_{R}{\mathrm{CD}}_{T}^{H}-D\right)\right)+\frac{\rho }{2}||B-D_{R}{\mathrm{CD}}_{T}^{H}-D||{}_{F}^{2} \end{array}$$

In Equation (8), $Z_{1}{\;\mathrm{and}\;Z}_{2}\in {\mathbb{C}}^{N_{R}\times N_{T}}$ are assigned as Lagrange multipliers also known as dual variables. On the other side ρ is contemplated as the step size of the algorithm and it always been consider as a positive integer. For the better understanding of S-ADMM, the ADMM is described first and then the symmetrical version is discussed on the base of ADMM. ADMM is already used to solve the cost function described in Equation (8) [33] and it generates its order as follows:

(9) $$A^{\left(l+1\right)}=\underset{A}{\mathrm{argmin}ℒ}\left(A,,B^{\left(l\right)},C^{\left(l\right)},D^{\left(l\right)},Z_{1}^{\left(l\right)},Z_{2}^{\left(l\right)}\right)$$

(10) $$B^{\left(l+1\right)}=\underset{B}{\mathrm{argmin}}ℒ\left(A^{\left(l+1\right)},B,C^{\left(l\right)},D^{\left(l\right)},Z_{1}^{\left(l\right)},Z_{2}^{\left(l\right)}\right)$$

(11) $$C^{\left(l+1\right)}=\underset{C}{\mathrm{argmin}}ℒ\left(A^{\left(l+1\right)},B^{\left(l+1\right)},C,D^{\left(l\right)},Z_{1}^{\left(l\right)},Z_{2}^{\left(l\right)}\right)$$

(12) $$D^{\left(l+1\right)}=\underset{D}{\mathrm{argmin}}ℒ\left(A^{\left(l+1\right)},B^{\left(l+1\right)},C^{\left(l+1\right)},D,Z_{1}^{\left(l\right)},Z_{2}^{\left(l\right)}\right)$$

(13) $$Z_{1}^{\left(l+1\right)}=Z_{1}^{\left(l\right)}+\rho \left(A^{\left(l+1\right)}-B^{\left(l+1\right)}\right)$$

(14) $$Z_{2}^{\left(l+1\right)}=Z_{2}^{\left(l\right)}+\rho \left(B^{\left(l+1\right)}-D_{R}C^{\left(l+1\right)}D_{T}^{H}-D^{\left(l+1\right)}\right)$$

The optimal solution of above equations can be obtained very easily as the main targeted cost function gets scattered in to 6 sub parts. Therefore, by following ways the variable described in Equations (9)–(14) can be solved,

#### 3.2.1. Solution of A

The closed-form solution $A^{l+1}$ is determined by considering all the terms related to A in Equation (8) and implementing SVT [41] on them. Therefore:

(15) $$ℒ≜\underset{A}{\mathrm{argmin}}\tau_{A}{\left||A|\right|}_{*}+\mathrm{tr}\left(Z_{1}^{H}\left(A-B\right)\right)+\frac{\rho }{2}||A-B||{}_{F}^{2}\;=\;\tau_{A}{\left||A|\right|}_{*}+\frac{\rho }{2}||A-{\left(B^{\left(l\right)}-\frac{1}{\rho }Z_{1}^{\left(l\right)}\right)||}_{F}^{2}$$

(16) $$A^{\left(l+1\right)}\;=\;\mathrm{Udiag}({\left\{\mathrm{sign}\left(h_{i}\right)\max\left(h_{i},0\right)\right\}}_{1\leq i\leq r})V^{H}$$

Here, $U\in {\mathbb{C}}^{N_{r}\times r}\;\mathrm{and}\;V\in {\mathbb{C}}^{N_{r}\times r}$ are the side singular vector of the matrices $\left(B^{\left(l\right)}-\frac{1}{\rho }Z_{1}^{\left(l\right)}\right)$ and $h_{i}≜\mu_{i}-\frac{\tau }{\beta }$. τ is known as SVT operator and the r singular values are denoted by $\mu_{i}$.

#### 3.2.2. Solution of B

For the close form solution of $B^{\left(l+1\right)}$, we consider all the terms related to B in Equation (8) and set it to the zero. Thus:

(17) $$ℒ≜\underset{B}{\arg\;\min}\frac{1}{2}||\Psi \circ B-A_{\Psi }||{}_{F}^{2}+\mathrm{tr}\left(Z_{1}^{H}\left(A-B\right)\right)+\frac{\rho }{2}||A-B||{}_{F}^{2}+\mathrm{tr}\left(Z_{2}^{H}\left(B-D_{R}{\mathrm{CD}}_{T}^{H}-D\right)\right)\;\;+\frac{\rho }{2}||B-D_{R}\mathrm{CD}-D||{}_{F}^{2}$$

$$ℒ\;=\;\Psi \circ B-A_{\Psi }-Z_{1}-\rho \left(A-B\right)-Z_{2}-\rho \left(D-B+D_{R}{\mathrm{CD}}_{T}^{H}\right)$$

$$\Psi \circ B-A_{\Psi }-Z_{1}-\rho \left(A-B\right)-Z_{2}-\rho \left(D-B+D_{R}{\mathrm{CD}}_{T}^{H}\right)\;=\;0$$

$$\Psi \circ A+2\rho B\;=\;{\;A}_{\Psi }+Z_{1}+\;\rho \left(A\right)+\;Z_{2}+\;\rho D+D_{R}{\mathrm{CD}}_{T}^{H}$$

(18) $$B\;=\;{\left(G+2\;\rho I\right)}^{-1}(Z_{1}+\;\rho \left(A\right)+\;Z_{2}+\;\rho D+\rho HC)$$

Here, I illustrate the identity matrix, whereas G$≜\overset{N_{R}}{\underset{i=1}{\sum }}\mathrm{diag}{\left({\left[\Psi \right]}_{k}\right)}^{T}⨂E_{\mathrm{kk}}$ ${\left[\Psi \right]}_{i}$, exhibits the k-th row, and $E_{\mathrm{kk}}$ is derived by inserting unit values in the $N_{R}\times N_{R}$ zero matrix at its (k,k)-th position as well as H$≜D_{T}^{*}⨂D_{R}$ [33,34].

Hence, for (l + 1) iteration of b is:

(19) $$b^{\left(l+1\right)}\;=\;{\left(G^{H}G+2\rho I\right)}^{-1}\left(z_{1}^{\left(l\right)}+{\rho a}^{\left(l+1\right)}+G^{H}a_{\Psi }+z_{2}^{\left(l\right)}+{\rho d}^{\left(l\right)}+{\rho \mathrm{Hc}}^{\left(l\right)}\right)\;$$

For $B^{\left(l+1\right)}$ unvectorized Equation (19), thus:

(20) $${\;B}^{\left(l+1\right)}\;=\;\mathrm{unvec}\left(b^{\left(l+1\right)}\right)$$

#### 3.2.3. Solution of C

For the close form solutions of $C^{l+1}$, separate all the term of C in Equation (8). Therefore:

(21) $$ℒ≜\underset{C}{\mathrm{argmin}}\;\tau_{C}{\left||C|\right|}_{1}+\mathrm{tr}\left(Z_{2}^{H}\left(B-D_{R}{\mathrm{CD}}_{T}^{H}-D\right)\right)+\frac{\rho }{2}||B-D_{R}{\mathrm{CD}}_{T}^{H}-{D||}_{F}^{2}\;\;\;={\;\tau }_{C}{\left||C|\right|}_{1}+\frac{\rho }{2}{\left||D_{R}^{H}\left(\frac{1}{\rho }Z_{2}^{\left(l+1\right)}-D^{\left(l\right)}+B^{\left(l+1\right)}\right)D_{T}|\right|}_{F}^{2}$$

Here, Equation (21) is considered as the standard least absolute shrinkage and selection operator (LASSO) problem [50]. Therefore, to solve Equation (21), vectorization is performed:

(22) $$\underset{c}{\mathrm{argmin}}\tau_{C}{\left||C|\right|}_{1}+\frac{\rho }{2}{\left||D_{R}^{H}\left(\frac{1}{\rho }Z_{2}^{\left(l+1\right)}-D^{\left(l\right)}+B^{\left(l+1\right)}\right)D_{T}|\right|}_{F}^{2}$$

Let us consider, $J^{\left(l+1\right)}\;={\;D}_{R}^{H}D_{T}\left(\frac{1}{\rho }(Z_{2}^{\left(l+1\right)}-D^{\left(l+1\right)}+B^{\left(l+1\right)}\right)$ and $j^{\left(l+1\right)}\;=\;\mathrm{vec}\left(J^{\left(l+1\right)}\right)$. Hence, Equation (22) can be equivalently expressed as:

(23) $$\underset{c}{\mathrm{argmin}}\tau_{c}{\left||C|\right|}_{1}+\frac{\rho }{2}||c-j^{\left(l+1\right)}||{}_{F}^{2}$$

Afterwards, a soft thresholding operator is applied on Equation (23) for (l + 1) iterations:

(24) c(l+1)=sign(Re(j(l+1)))∘max(|Re(j(l+1)|−τc′,0)+sign(Im(j(l+1)))∘max(|Im(j(l+1)|−τc′,0)

Here, $\tau_{c}^{\prime }$ is known as the scaled version of $\tau_{c}$ and $\tau_{c}^{\prime }≜\frac{\tau_{c}}{\rho }$. Therefore, $C^{\left(l+1\right)}$ is obtained by unvectorizing the $c^{\left(l+1\right)}$:

(25) $$C^{\left(l+1\right)}\;=\;\mathrm{unvec}\;(c^{\left(l+1\right)})$$

#### 3.2.4. Solution of D

To get the solution of $D^{l+1}$, we consider all the terms related to D in Equation (8) and set them to zero:

$$ℒ≜\underset{D}{\mathrm{argmin}}\frac{1}{2}{\left||D|\right|}_{F}^{2}+\left(Z_{2}^{H}\left(B-D_{R}{\mathrm{CD}}_{T}^{H}-D\right)\right)+\frac{\rho }{2}||B-D_{R}{\mathrm{CD}}_{T}^{H}-D||{}_{F}^{2}$$

$$ℒ\;=\;(1+\rho )D-\rho \left(B-D_{R}\mathrm{CD}+\frac{Z_{2}^{H}}{\rho }\right)$$

Therefore, the solution of $D^{\left(l+1\right)}$ can be expressed as:

(26) $$D^{\left(l+1\right)}\;=\;\frac{\rho }{\rho +1}\left(B^{\left(l+1\right)}-D_{R}C^{\left(l+1\right)}D_{T}^{H}+\frac{1}{\rho }Z_{2}^{\left(l\right)}\right)$$

The solutions of A, B, C and D can update the Equations (9)–(14). Subsequently, according to Langrage multiplies methods, the dual variables $Z_{1}$ and $Z_{2}$ can be update with help of the A, B, C and D’s solutions.

Algorithm 1 describes the channel estimation method of a mmWave MIMO system via ADMM. Here, the intermediate and essential variable are updated first and the dual variables are updated at the last. As described in [51], Fortin and Glowinski proposed that, attaching a relaxation parameter in ADMM lead to the faster convergence. Therefore, according to the Fortin and Glowinski proposed idea Equations (9)–(14) can be written as:

(27) $$A^{\left(l+1\right)}\;=\;\underset{A}{\mathrm{argmin}ℒ}\left(A,,B^{\left(l\right)},C^{\left(l\right)},D^{\left(l\right)},Z_{1}^{\left(l\right)},Z_{2}^{\left(l\right)}\right)$$

(28) $$B^{\left(l+1\right)}=\underset{B}{\mathrm{argmin}}ℒ\left(A^{\left(l+1\right)},B,C^{\left(l\right)},D^{\left(l\right)},Z_{1}^{\left(l\right)},Z_{2}^{\left(l\right)}\right)$$

(29) $$C^{\left(l+1\right)}=\underset{C}{\mathrm{argmin}}ℒ\left(A^{\left(l+1\right)},B^{\left(l+1\right)},C,D^{\left(l\right)},Z_{1}^{\left(l\right)},Z_{2}^{\left(l\right)}\right)$$

(30) $$D^{\left(l+1\right)}=\underset{D}{\mathrm{argmin}}ℒ\left(A^{\left(l+1\right)},B^{\left(l+1\right)},C^{\left(l+1\right)},D,Z_{1}^{\left(l\right)},Z_{2}^{\left(l\right)}\right)$$

(31) $$Z_{1}^{\left(l+1\right)}=Z_{1}^{\left(l\right)}+\alpha \rho \left(A^{\left(l+1\right)}-B^{\left(l+1\right)}\right)$$

(32) $$Z_{2}^{\left(l+1\right)}=Z_{2}^{\left(l\right)}+\alpha \rho \left(B^{\left(l+1\right)}-D_{R}C^{\left(l+1\right)}D_{T}^{H}-D^{\left(l+1\right)}\right)$$

**Algorithm 1. mmWave MIMO Channel Estimation Scheme via ADMM** [33]***Require*:**Subsampled matrix $A_{\Psi }$, side information matrices $D_{R}$ and $D_{T}$, and the set of indices of observed entries in Ψ.***Input*:**$A_{\Psi }$, Ψ,${\;D}_{R}$, $D_{T}$, ρ, $\tau_{A}$,${\;\tau }_{C}$ and $I_{\max}$
***Output*:**Estimated output channel matrix $\hat{A}\;=\;A^{\left(I_{max}\right)}$ **Initialization:**        $A^{\left(0\right)}\;={\;B}^{\left(0\right)}\;={\;C}^{\left(0\right)}\;={\;D}^{\left(0\right)}\;={\;Z}_{1}^{\left(0\right)}\;={\;Z}_{2}^{\left(0\right)}\;=\;0$
***Step 1*:****for** l = 0, 1, 2……. $I_{\max}-1$
***Step 2*:**Update ${\;A}^{\left(l+1\right)}$ by using Equation (16).***Step 3*:**Update $B^{\left(l+1\right)}$ by using the Equation (20).***Step 4*:**Update $C^{\left(l+1\right)}$ by using the Equation (25).***Step 5*:**Update $D^{\left(l+1\right)}$ by using the Equation (26).***Step 6*:**Update $Z_{1}^{\left(l+1\right)}{\;\mathrm{and}\;Z}_{1}^{\left(l+1\right)}$ by using Equations (13) and (14), respectively.***Step7*:****end for**

Here, α is known as Fortin and Glowinski’s relaxation parameter. Typically, α rely in between 0 and $\frac{1+\sqrt{5}}{2}$, which can be approximated around 0 and 2. Multiplication of relaxation parameter α with the ρ is enlarge the step size of ADMM and lead to the faster convergence [51,52,53].

ADMM defined in Equations (27)–(32) is different than the ADMM defined in Equations (9)–(14). To all intents and purposes, there are two distinct families of ADMM, one is derived from the operator splitting framework and the other derived from the Lagrangian splitting [54]. Therefore, except for the notation similarity of the ADMM defined in Equations (9)–(14) and Equations (27)–(32), the ADMM scheme with Fortin and Glowinski’s relaxation parameter is different than the ADMM define in Equations (9)–(14) in nature [35].

As observed in [55,56], the ADMM scheme described in Equations (9)–(14) is an application of the Douglas–Rachford splitting method (DRSM) in [57,58] to the dual of Equations (9)–(14). However, If the Peaceman–Rachford splitting method (PRSM), which is described in [58,59] is implemented on ADMM described in Equations (27)–(32), the resultant new ADMM can be expressed as:

(33) $$A^{\left(l+1\right)}=\underset{A}{\mathrm{argmin}ℒ}\left(A,,B^{\left(l\right)},C^{\left(l\right)},D^{\left(l\right)},Z_{1}^{\left(l\right)},Z_{2}^{\left(l\right)}\right)$$

(34) $$Z_{1}^{\left(l+\frac{1}{2}\right)}=Z_{1}^{\left(l\right)}+\alpha \rho \left(A^{\left(l+1\right)}-B^{\left(l\right)}\right)$$

(35) $$Z_{2}^{\left(l+\frac{1}{2}\right)}=Z_{2}^{\left(l\right)}+\alpha \rho \left(B^{\left(l\right)}-D_{R}C^{\left(l\right)}D_{T}^{H}-D^{\left(l\right)}\right)$$

(36) $$B^{\left(l+1\right)}=\underset{B}{\mathrm{argmin}}ℒ\left(A^{\left(l+1\right)},B,C^{\left(l\right)},D^{\left(l\right)},Z_{1}^{\left(l\right)},Z_{2}^{\left(l\right)}\right)$$

(37) $$C^{\left(l+1\right)}=\underset{C}{\mathrm{argmin}}ℒ\left(A^{\left(l+1\right)},B^{\left(l+1\right)},C,D^{\left(l\right)},Z_{1}^{\left(l\right)},Z_{2}^{\left(l\right)}\right)$$

(38) $$D^{\left(l+1\right)}=\underset{D}{\mathrm{argmin}}ℒ\left(A^{\left(l+1\right)},B^{\left(l+1\right)},C^{\left(l+1\right)},D,Z_{1}^{\left(l\right)},Z_{2}^{\left(l\right)}\right)$$

(39) $$Z_{1}^{\left(l+1\right)}=Z_{1}^{\left(l+\frac{1}{2}\right)}+\alpha \rho \left(A^{\left(l+1\right)}-B^{\left(l+1\right)}\right)$$

(40) $$Z_{2}^{\left(l+1\right)}=Z_{2}^{\left(l+\frac{1}{2}\right)}+\alpha \rho \left(B^{\left(l+1\right)}-D_{R}C^{\left(l+1\right)}D_{T}^{H}-D^{\left(l+1\right)}\right)$$

The above described scheme in Equations (33)–(40) is known as symmetrical ADMM (S-ADMM) wherein, all the variables are treated in a symmetrical manner.

### 3.3. Algorithm Elucidation

The proposed S-ADMM scheme for the channel estimation of a mmWave MIMO system is described in Algorithm 2. Within this scheme, the matrix Ψ has the non-zero uniformly distributed entries at their respective ij-th position in such a way that $\Psi \;=\;\left\{1,2,3,\ldots N_{R}N_{T}\right\}$ [60,61]. Notably, these non-zero values are chosen in a haphazard manner. Therefore, it can be argued that the matrix Ψ has M ones and $\left(N_{R}N_{T}-M\right)$ zeros. Matrix Ψ is followed by a subsampled matrix $A_{\Psi }$. Thus, the entries of $A_{\Psi }$ is also followed by the entries of Ψ., so the positions of non-zero entries in $A_{\Psi }$ are also similar to the positions on non-zero entries in Ψ. The error caused during the estimation of A depends upon the estimation accuracy of $A_{\Psi }$’s elements and the M non-zero values of $A_{\Psi }$ [34]. The threshold point, where the training symbols length are equal to the position of the non-zero entries in A i.e., T = M and M $\ll N_{R}N_{T}$, is considered as a stopping criterion for the proposed S-ADMM scheme.
**Algorithm 2. Proposed S-ADMM based mmWave MIMO Channel Estimation Scheme*****Require*:**Subsampled matrix $A_{\Psi }$, side information matrices $D_{R}$ and $D_{T}$, and the set of indices of observed entries in Ψ.***Input*:**$A_{\Psi }$, Ψ,${\;D}_{R}$, $D_{T}$, ρ, $\alpha ,{\;\tau }_{A}$,${\;\tau }_{C}$ and $I_{\max}$
***Output*:**Estimated output channel matrix $\hat{A}\;=A^{\left(I_{max}\right)}$ **Initialization:**        $A^{\left(0\right)}={\;B}^{\left(0\right)}={\;C}^{\left(0\right)}={\;D}^{\left(0\right)}=Z_{1}^{\left(0\right)}={\;Z}_{2}^{\left(0\right)}=0$
***Step 1*:****for** l = 0, 1, 2……. $I_{\max}-1$
***Step 2*:**Update ${\;A}^{\left(l+1\right)}$ by using Equation (33) and it gets updated from the solution in Equation (16)***Step 3*:**Update $Z_{1}^{\left(l+\frac{1}{2}\right)}$ and $Z_{2}^{\left(l+\frac{1}{2}\right)}$ by using Equations (34) and (35), respectively.***Step 4*:**Update $B^{\left(l+1\right)}$ by using the Equation (36) and it used solution described in Equation (20)***Step 5*:**Update $C^{\left(l+1\right)}$ by using the Equation (37) and the solution of C is updated by Equation (25).***Step 6*:**Update $D^{\left(l+1\right)}$ by using the Equation (38) and the solution of D is provided by Equation (26).***Step 7*:**Update $Z_{1}^{\left(l+1\right)}{\;\mathrm{and}\;Z}_{1}^{\left(l+1\right)}$ by using Equations (39) to (40), respectively.***Step8*:****end for**

### 3.4. Complexity Analysis

In the proposed S-ADMM scheme, step 2 is the most important and decisive part. In step 2, SVT operator is implemented on the non-squared matrix A. The SVT is nothing but another version of singular value decomposition (SVD), where the targeted matrix is transformed in to an orthogonal matrix to ensure orthogonality. Therefore, the order of complexity required to compute the step 2 is proportional to $M_{T}^{2}$ [62]. Step 4 of the proposed scheme has the solutions of the Equation (18) is illustrated by the inversion of G+2 ρI. However, this matrix is a diagonal matrix, therefore, the required complexity is $O(TN_{R})$. In step 6, the pseudo-inverse of $H\in {\mathbb{C}}^{{\mathrm{TN}}_{R}\times L_{P}N_{T}N_{R}}$ has to be calculated which needs the calculation and conversion of the Gram matrix $H^{H}H\in {\mathbb{C}}^{LN_{T}N_{R}\times LN_{T}N_{R}}$ [16]. However, this step is the very expensive and cost huge computational load. Nonetheless, $H^{H}H$ is already noted as a presiding diagonal matrix, hence gradient-based iterative algorithms is used to lower the complexity order to $O(L\;N_{T}N_{R})$ [63]. The rest of the steps are acting as a matrix- matrix and matrix- vector products, which inherently needs lower computational power.

## 4. Simulation and Results

In this section, simulations are carried out and results are explained in detail. To illustrate the preeminence of the proposed S-ADMM scheme, by considering the parameters with their respective values listed in Table 2, a simulation is performed and the detailed results are explained.

Seven different state-of-art benchmark algorithms namely: OMP, VAMP, ADMM, Ex-ADMM, GAMP-GM, BOMP and SVT are taken into account for the comparison with proposed S-ADMM algorithm. The basic and working methodology of all five benchmark algorithms are entirely different from each other which is the main motivation to consider them for performance comparison with our proposed scheme. The performance of the proposed S-ADMM scheme is compared with these benchmarks in term of NMSE, ASE, convergence and effects of scatterers as well as with the number of paths.

### 4.1. NMSE Comparison

To demonstrate the performance of S-ADMM in terms of NMSE, low training symbol lengths i.e., T = 400 and high training symbol lengths T = 1200, is considered for simulation. The relation is used to calculate the NMSE is described as follows:

(41) $$\mathrm{NMSE}≜E\left(10\log_{10}\frac{||\hat{A}-{A||}_{F}^{2}}{{\left||A|\right|}_{F}^{2}}\right)\;$$

**Figure 2:**
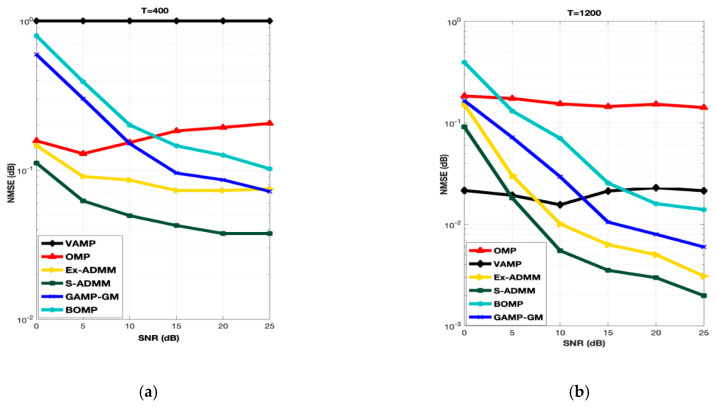
NMSE performance of S-ADMM for T = 400 (**a**) and T = 1200 (**b**) at 30 dB SNR.

Performance of OMP is moderated at low SNR points (i.e., <5 dB) but as the SNR is increasing from low to mid and then to the high the performance is started decreasing. This happens due to the discretization error caused in dictionary matrix. GAMP-GM approximate any vector in to a scaler which reduce the complexity of the algorithm and an enhancement in performance is beheld. Hence, it is clear from Figure 2 that the performance of GAMP-GM is unquestionably better than that of another approximate message passing algorithm VAMP for small and high training symbol lengths. For T = 400 and 1200, at low- to mid-SNR points, the performance of GAMP-GM is very significant but as the SNR range is increased from mid to high, the performance is slightly getting worse. The reason behind this is that the GAMP-GM diverges with the overcomplete dictionary matrices resulting from beam domain quantization. BOMP is another popular basic pursuit algorithm for the recovery of sparse signals which exhibits the additional structure in the form of the nonzero coefficients occurring in channel matrix. Such signals are referred to as block-sparse. In Figure 2, for T = 400 at low to mid SNR points, the performance of BOMP is trail behind the OMP but as the SNR range is increased from mid to high the performance of BOMP is getting much better as compare to OMP. For T = 1200, BOMP outperformed OMP because OMP is not capable enough to recover the large training symbols. This happens as the BOMP exploits the block sparse structure of channel matrix. Therefore, large and small training symbols can be recovered by using BOMP. Practically, in BOMP, the spatial frequencies corresponding to the AoA and AoD of each path may not fall exactly in the grid points of DFT matrices of ULA size which caused the performance degradation. The Ex-ADMM is an extension of ADMM and it performs well at almost all SNR points. Although, the performance of the S-ADMM is better than all other benchmark algorithms. The reason behind that is, the Ex-ADMM only use relaxation factor to enlarge the step size but the S-ADMM make the step size enlarger in addition with the symmetrical treatment for all variables. When high training symbols length i.e., T = 1200 are chosen for simulation, VAMP improved its performance at almost all SNR points. On the contrary, the performance of OMP remain in the same condition due to the fact that, at high SNR and large training symbols length, OMP suffered from discretization error and it is not capable to recover the transmitted symbols properly. Ex-ADMM keep performing at T = 1200 mid to high SNR points but it is underperformed by S-ADMM.

### 4.2. ASE Comparison

For the performance evaluation in terms of ASE of proposed S-ADMM scheme, similar as NMSE, low (T = 400) and high (T = 1200) training symbols lengths have been considered for simulation. The relation assigned to calculate the ASE [65,66] is:

(42) $$\mathrm{ASE}=\;E\{\log_{2}\det(I_{N_{R}}+\left(N_{R}N_{T}{\left(\sigma_{q}^{2}+\mathrm{NMSE}\right)}^{-1}{\mathrm{AA}}^{H}\right)\}$$

**Figure 3:**
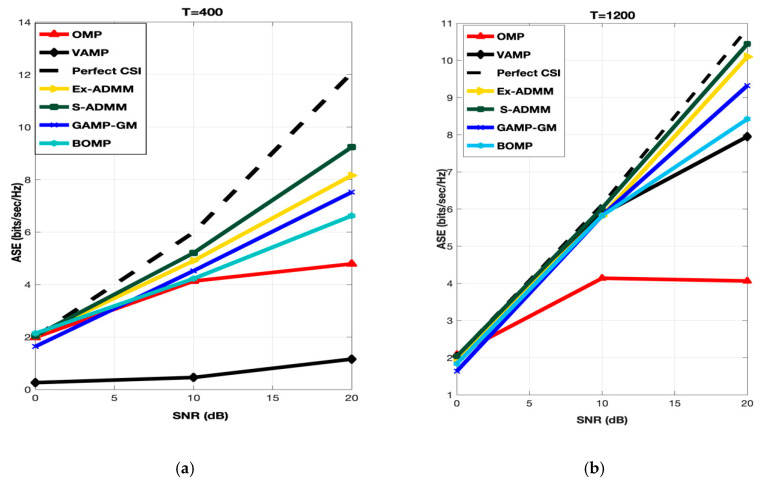
ASE performance of S-ADMM at T = 400 (**a**) and T = 1200 (**b**).

Figure 3 explains the performance evaluation of the proposed S-ADMM scheme in comparison with OMP, VAMP, BOMP, GAMP-GM and Ex-ADMM. In the case of OMP, for T = 400 in all the SNR range, it performed ordinarily but the performance of OMP is getting worse as the length of training lengths as well as the SNR range are increasing. For T = 400 at low to mid SNR range the performance of GAMP-GM is nearly similar to Ex-ADMM and BOMP but as the SNR range is increased from mid to high, GAMP-GM outperformed BOMP, OMP and VAMP. For T = 1200 at low to mid SNR range, GAMP-GM are very close to VAMP, Ex-ADMM, BOMP and proposed S-ADMM. As the SNR points are increasing from mid to high, the performance of GAMP-GM is improving linearly and it outperformed the VAMP, BOMP and OMP. In case of BOMP, for T = 400 and 1200 at low to mid SNR range, it performed better than the OMP and VAMP. Same pattern is observed at mid to high SNR range. Therefore, one can see that, the performance of BOMP is better than the OMP and VAMP for all SNR points as well as for all training symbol lengths. As discussed earlier, VAMP is not designed for low training symbols therefore it performed worst for T = 400 at almost every SNR range. For high training symbols length (i.e., T = 1200), VAMP shows a significant improvement and performed very well from low to mid as well as from mid to high SNR points. As a matter of fact, VAMP outperformed OMP at all SNR points. Interestingly, the performance of the proposed S-ADMM and Ex-ADMM are almost equal at all SNR points. However, the performance of proposed S-ADMM is slightly improved as compared to Ex-ADMM. In conclusion, the proposed S-ADMM outperformed all other benchmark algorithms at all SNR points for low training symbols length.

### 4.3. Comparison of Convergence

**Figure 4:**
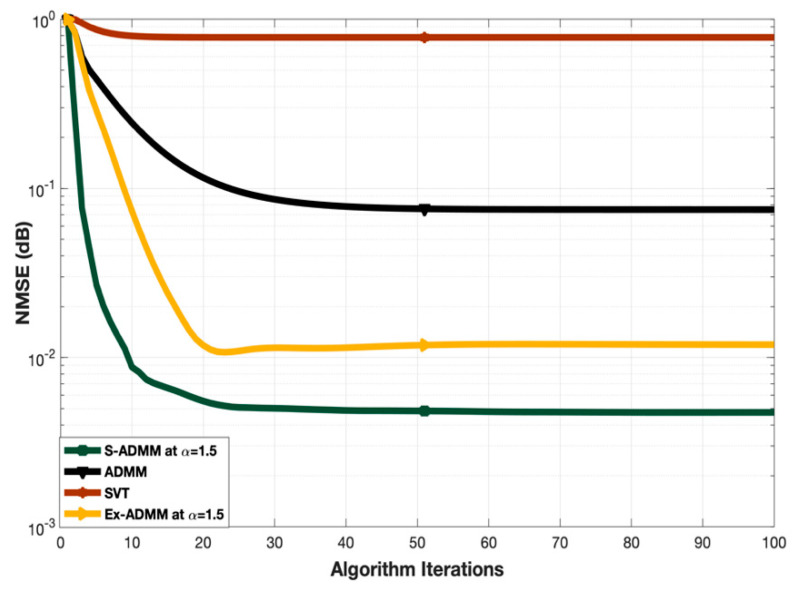
Convergence comparison at α = 1.5.

Figure 4 illustrates the convergence of the proposed S-ADMM scheme. In order to compare the convergence, benchmarks SVT, ADMM and Ex-ADMM have been considered. The number of training symbols is fixed at 400 as well as the relaxation parameter α for Ex-ADMM and proposed S-ADMM is set to be 1.5. As one can see from Figure 4, the SVT converges fast as it is a one stage direct method but its NMSE performance is worse than all of the other benchmarks and the proposed S-ADMM. The convergence of ADMM and Ex-ADMM is almost identical and moderate but the NMSE performance of Ex-ADMM is better than that of ADMM. Convergence of the proposed S-ADMM is better than that of all other benchmarks and its NMSE performance also outperforms the others. Eventually, the SVT started converging around 5–10 iterations. The ADMM and Ex-ADMM take around 15–20 iterations to converge. However, the proposed S-ADMM started converging around 7–12 iterations. Therefore, one can observe that the proposed S-ADMM outperformed all other state-of-art benchmark algorithms in terms of convergence which makes it faster than all the other described methods.

### 4.4. Effect on Number of Scatterers and Paths

**Figure 5:**
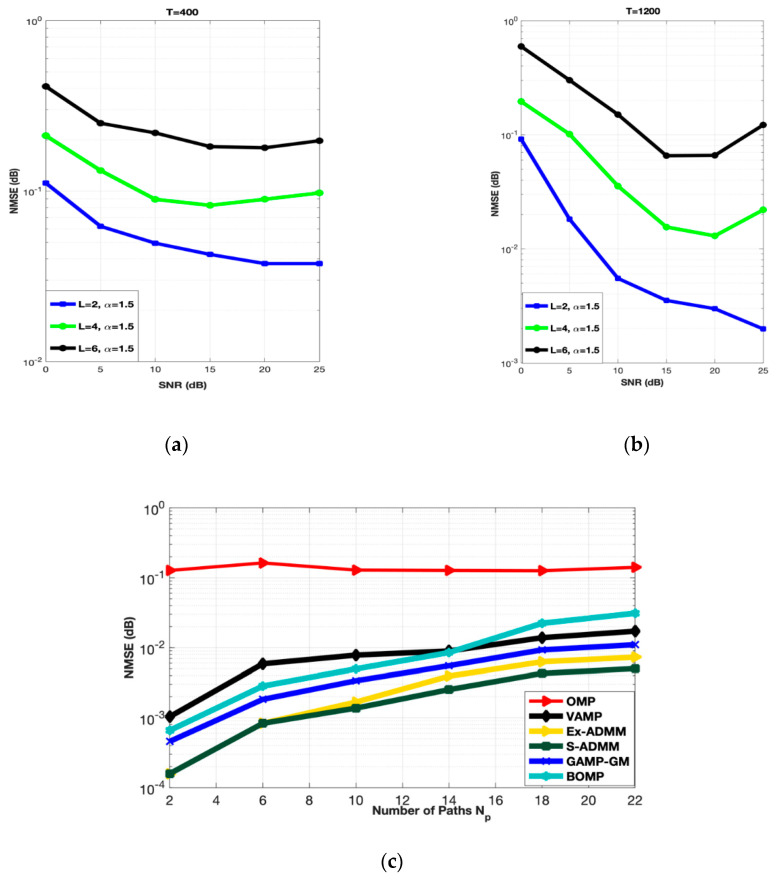
(**a**,**b**) Number of scatterers vs. NMSE at 30 dB SNR. (**c**) Number of paths vs. NMSE.

Figure 5 elaborates the performance of the proposed S-ADMM scheme over several scatterers and the number of paths. It is observed that as the number of scatterers and number channel paths is inversely proportional to the NMSE performance of the system.

Figure 5a demonstrates the performance of the proposed S-ADMM scheme for low training symbols length (i.e., T = 400). The number of scatterers is set to be L = 2, 4 and 6, respectively. All the results are obtained at α = 1.5. It can be observed that the NMSE performance of the proposed S-ADMM scheme is getting worse as the number of scatterers are increasing. The same thing happened in the case of high training symbols length (i.e., T = 1200). Figure 5b also indicates the same results that as the number of scatterers are increasing the NMSE performance is getting worse. This happens because of the worse scattering nature of mmWaves. Figure 5c depicts the effects of escalation in path of mmWaves. Therefore, to summarize, by the inherent nature of mmWave, it can be said that the performance of a mmWave MIMO system decreases as the number of propagation paths and scatterers increases, but still the performance of the proposed S-ADMM is much better than the OMP, VAMP, GAMP-GM, BOMP and the Ex-ADMM as depicted in Figure 5c.

## 5. Concluding Remarks

To jointly optimize the low rank and sparsity-based problem for the channel estimation of a mmWave MIMO system, a symmetrical version of ADMM (S-ADMM) has been proposed. The S-ADMM treated every variable symmetrically in the optimization problem. For better convergence rate, to enhance the step size, a relaxation parameter is multiplied into the step size of duals. In order to get better optimal solutions, the proposed scheme divides the optimization problems into several subproblems and solves them individually. Although, S-ADMM is better for recovering the training symbols, the performance is degraded when the number of scatterers and paths are increased. Therefore, there is room for improvement. With proper modifications, the proposed S-ADMM algorithm can be further extended for the estimation of time-varying mmWave channels in a hybrid MIMO system. Extensive simulations experiments are carried out to explain the validation and superiority of the scheme. Comprehensively, the proposed S-ADMM scheme performed better than all other state-of-art benchmark algorithms considered in this work.

## Figures and Tables

**Table 1:** Notation.

α,a and A	Scaler, vector and matrix.
$A^{T},A^{H}{\;\mathrm{and}\;A}^{*}$	Matrix transpose, conjugate transpose and conjugate.
${\left||\left(.\right)|\right|}_{F}$,$\;{\left||\left(.\right)|\right|}_{*}$ and ${\left||\left(.\right)|\right|}_{1}$	Frobenius norm, nuclear norm and $l_{1}-\mathrm{norm}$
Operands ∘and ⊗	Matrix Hadamard and Kronecker products.
vec (.)	Vectorization of (.).
unvec (.)	Inverse operation of vec(.).
E{.}	Expected value of {.}.
diag(.)	Diagonal of (.).
$I_{N}$	N × N identity matrix.

**Table 2:** Simulation Parameters.

Carrier Frequency	90 GHz
Maximum numbers of iterations	100 [64]
Maximum numbers of Monte Carlo realizations	100 [64]
Number of transmitter antennas	64
Number of transmitter antennas	64
Spacing between antennas d	$\frac{\lambda }{2}$
Signal-to-noise Ratio (SNR) $\sigma_{q}^{-2}$	30 dB
Number of mmWave channel path	2
Number of clusters	1
Standard deviation of uniformly distributed AoA’s and AoD’s	55°
Uniform distribution range of AoA’s and AoD’s	[0, 2π]
Relaxation factor	1.5
Weighting factors	$\tau_{A}=\rho ||A_{\psi }||$ and $\tau_{C}=\frac{0.1}{\left(1-10\log\left(\sigma_{q}^{2}\right)\right)}$
Step size	$\rho =\frac{3M}{N_{R}N_{T}}\;$= 0.005
